# Effects of Reduced Prolamin on Seed Storage Protein Composition and the Nutritional Quality of Rice

**DOI:** 10.3390/ijms140817073

**Published:** 2013-08-19

**Authors:** Hyun-Jung Kim, Jong-Yeol Lee, Ung-Han Yoon, Sun-Hyung Lim, Young-Mi Kim

**Affiliations:** Department of Agricultural Biotechnology, National Academy of Agricultural Science, Rural Development Administration (RDA), 224 Suinro Gwonseon-gu, Suwon 441-707, Gyeonggi-do, Korea; E-Mails: khj5757@korea.kr (H.-J.K.); jy0820@korea.kr (J.-Y.L.); uhyoon@korea.kr (U.-H.Y.); limsh2@korea.kr (S.-H.L.)

**Keywords:** rice, seed storage protein, protein body, prolamin, RNA interference, amino acid analysis

## Abstract

Rice seed storage proteins accumulate in two types of protein body (PB-I and PB-II) that are nutrient sources for animals. PB-I is indigestible and negatively affects rice protein quality. To improve the nutritional value of rice seeds we are aiming to engineer the composition and accumulation of endogenous seed storage proteins. In this study we generated transgenic rice plants in which 13 kD prolamin genes were suppressed by RNA interference (13 kD pro-RNAi). Analysis based on qRT-PCR confirmed that the targeted 13 kD prolamins were markedly suppressed, and were compensated for by an increase in other storage proteins including 10 kD prolamin, glutelins, and chaperone proteins. The storage protein profiles further revealed that the levels of 13 kD prolamins were significantly reduced, while that of the glutelin precursor was slightly increased and the remaining storage proteins did not change. Amino acid analysis showed that the reduction of 13 kD prolamins resulted in a 28% increase in the lysine content relative to the wild type, indicating that the 13 kD pro-RNAi rice seeds are more nutritious. Furthermore, a reduction in the levels of 13 kD prolamins resulted in abnormal formation of PB-I, which was small and had no lamellar structure. These results suggest that alteration of prolamins can contribute to improving the nutritional quality of rice.

## 1. Introduction

Rice (*Oryza sativa* L.) is one of the most important cereal crops worldwide, and the major source of human nutrition and livestock feed in many countries. During seed maturation endosperm cells accumulate large amounts of storage proteins as a source of carbon and nitrogen for germinated seedlings [1]. The rice seed storage proteins include acid/alkaline-soluble glutelins, alcohol-soluble prolamins, and saline-soluble α-globulin [2–5]. Glutelins are major rice seed storage proteins, accounting for 60%–80% of the total seed protein content. They are encoded by 15 gene copies in the genome; these are classified into four subfamilies (GluA, GluB, GluC, and GluD) based on amino acid sequence similarity [6]. The prolamins comprise 20%–30% of the seed protein and are encoded by a multigene family of 34 gene copies [7–9] in three groups determined by their relative molecular weights (10, 13, and 16 kD), with the major group being the 13 kD species. The 13 kD prolamins are further classified as class I, II, or III, depending on the abundance of cysteine residues [10]. The α-globulin protein is encoded by a single gene, and accounts for 2%–8% of the total seed protein [11,12].

The seed storage proteins are synthesized at the rough endoplasmic reticulum (ER), translocated to the ER lumen, then transferred to separate intracellular compartments of the plant endomembrane system [3,5,13,14]. Prolamins are stored in spherical protein bodies (PBs) derived from the ER (referred to as PB-I), whereas glutelins and globulin are accumulated in irregularly-shaped PB-II bodies derived from the protein storage vacuole (PSV). PB-I is spherical in structure (diameter 1–2 μm), has a low electron density, and is surrounded by rough ER membranes having attached polysomes [5,13]. The PB-II is an irregularly shaped electron-dense structure 2–4 μm in diameter [3,12,13].

Because PBs have an unbalanced amino acid composition and are deficient in some essential amino acids, the protein content and its amino acid composition are important considerations in determining nutritional quality and usability for producers and consumers. Many recent studies have sought to improve the nutritional quality of rice through changes to the content of seed storage proteins. Low glutelin content mutants and transgenic seeds have been shown to have markedly reduced glutelin content, but the total protein content remains at levels similar to the wild type (WT) [15–18]. The reduction in glutelin content was compensated for by increased synthesis of prolamin and other storage proteins at both the mRNA and protein levels. Similarly, 13 kD Pro-less transgenic seeds [17] contained a much reduced content of 13 kD prolamins, but the content of glutelin and globulin was increased. These results suggest that the distribution of storage proteins in rice seed is regulated by a homeostatic mechanism for maintenance of total seed storage protein content as a nutritional source during seed germination and early seedling growth.

In previous research aimed at improving seed quality using genetic engineering we performed large-scale analysis of expressed sequence tags (ESTs) in immature and germinating rice seeds, and identified a highly expressed 13 kD prolamin as the major prolamin type in both stages [19]. We also generated transgenic rice plants expressing a glutelin RNAi vector and recombinant red fluorescent protein (RFP), in which suppression of expression of endogenous seed storage protein resulted in higher levels of accumulation of recombinant protein relative to the WT [20]. In the present study, we generated transgenic rice plants in which 13 kD prolamin genes were suppressed by RNA interference (RNAi). Our results revealed that 13 kD prolamin proteins were reduced in 13 kD pro-RNAi transgenic seeds, and that this was compensated for by an increase in other storage proteins. The reduction of 13 kD prolamins resulted in changes in amino acid composition and PB-I structure. Our results suggest that regulation of endogenous storage protein composition by 13 kD pro-RNAi is an important step forward in genetic engineering for nutritional improvement in rice grain quality.

## 2. Results and Discussion

### 2.1. Generation of Transgenic Rice and Analysis of Transcript Levels

**Figure 1:**
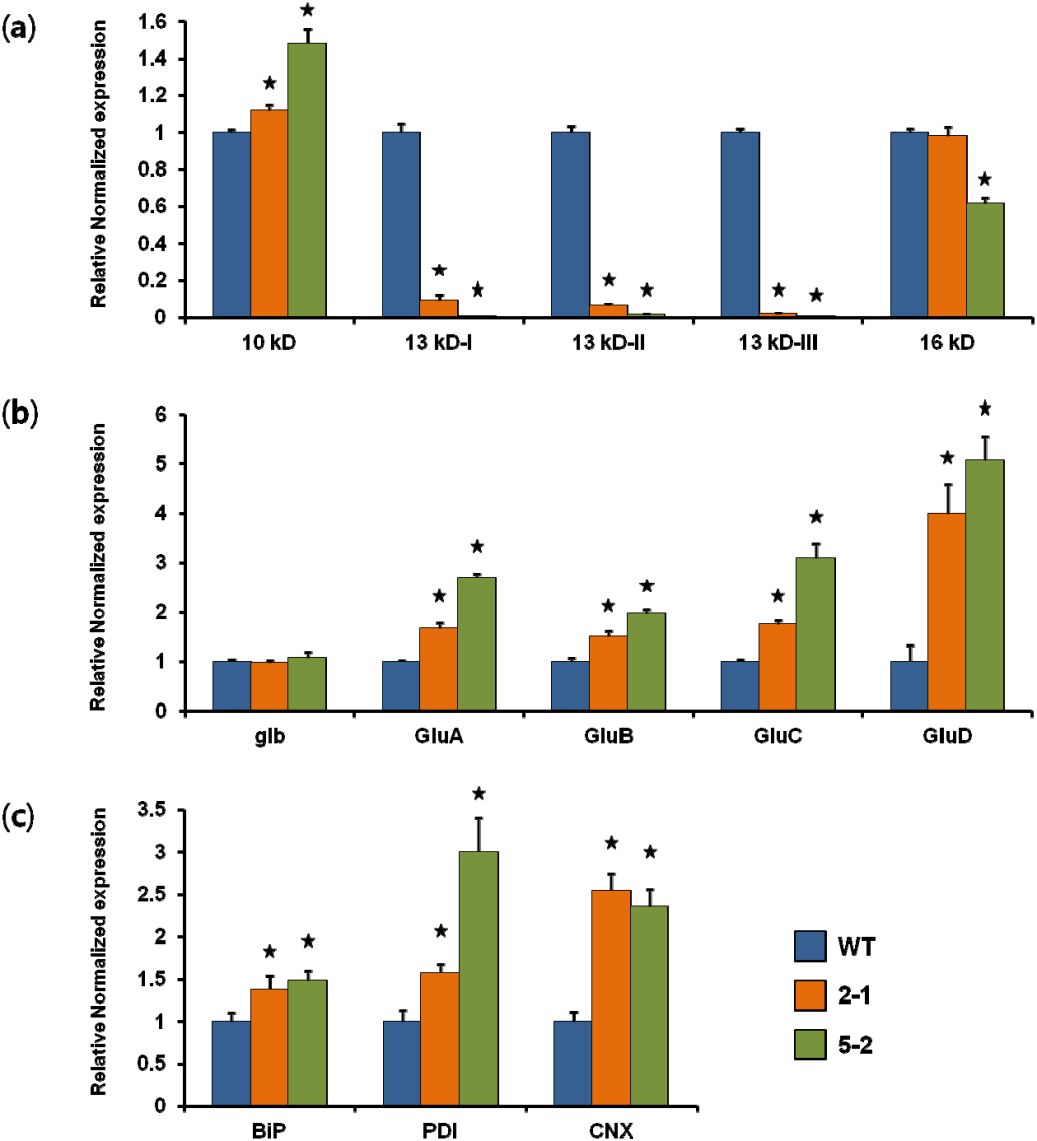
Transcript levels for the wild type (WT) and 13 kD pro-RNAi lines (2-1 and 5-2, respectively). Total RNA was extracted from immature seeds of WT or 13 kD pro-RNAi lines two weeks following flowering, reverse transcribed using oligo (dT) 15, and subjected to qRT-PCR using specific primers for prolamins (**a**), 10 kD, 13 kD-I, 13 kD-II, 13 kD-III and 16 kD; (**b**), α-globulin (glb), glutelins (GluA, GluB, GluC and GluD) and ER chaperone genes (**c**) BiP, PDI and CNX. The transcript levels were normalized to that of the internal control gene ubiquitin, and are represented relative to the expression levels in the WT. The values are means and SDs (error bars) of three replicates of the same RNA samples. The asterisks represent values significantly different from the wild type (*P* < 0.05). The primers are listed in Table S1.

Prolamins in rice are encoded by multigene families and expressed during immature and germinating seed stages. Three subgroups (classes I, II, and III) are recognized among the 13 kD prolamins, and these are expressed as major components in both developmental stages [19,21–23]. For efficient suppression of endogenous 13 kD prolamins the conserved region of the abundant EST clones (KCS140F05 and KCS204B08) was used to construct the RNAi cassette (13 kD pro-RNAi) [19]. The RNAi cassette was transferred into rice using *Agrobacterium*-mediated transformation. More than twenty independent transformation lines were initially selected using genomic PCR and southern blot analysis. The growth and morphology of the transgenic lines were apparently normal compared with the WT control. Two 13 kD pro-RNAi transgenic lines harboring a single copy of the 13 kD pro-RNAi gene were selected and self-pollinated to obtain homozygous lines. The transcript levels of the storage proteins were analyzed using qRT-PCR, based on total RNA extracted from immature seeds at two weeks after flowering (WAF). For prolamins the expression levels of the targeted 13 kD genes were markedly down-regulated in each of the 13 kD pro-RNAi transgenic lines. The suppression of 13 kD prolamin was compensated for by up-regulation of the 10 kD prolamin gene. In contrast, expression levels of the 16 kD gene were slightly decreased in only the 13 kD pro-RNAi 5-2 line of the selected transgenic lines (Figure 1a). Nucleotide similarity between trigger and target sequences is generally an important factor for gene suppression efficiency of RNAi. We reported that *Glu* RNAi transgenic rice with the glutelin A RNAi cassette was markedly suppressed in both GluA and GluB subfamily genes because of the high degree of similarity between the trigger sequence for *Glu* RNAi and the sequences in these gene subfamilies [20]. The nucleotide sequence similarities between 10 kD, 13 kD class II, 13 kD class III, and 16 kD to 13 kD class I prolamin genes are 39.0%, 78.5%, 82.0%, and 58.0%, respectively. Therefore, we suggest that down-regulation of the 16 kD prolamin gene in the 13 kD pro-RNAi 5-2 line is possibly a non-specific effect of the high similarity of the 16 kD prolamin gene sequences to the target sequence used for gene silencing.

For the other PB-II storage protein genes, glutelin gene expression was significantly up-regulated, but α-globulin expression was not changed in the 13 kD pro-RNAi lines. To investigate how transcript levels of glutelin subfamilies were reflected in the increased glutelin expression, we performed qRT-PCR using glutelin subfamily-specific primers designed to independently detect the transcript levels of gluA, gluB, gluC, and gluD (Table S1). The level of gluD was markedly increased in the 13 kD pro-RNAi lines, whereas the levels of GluA, GluB, and GluC were slightly increased (Figure 1b). Prolamin knockdown rice has been reported in several studies. For example, in 13 kD Pro-less transgenic rice seeds the targeted prolamin gene expression levels were effectively down-regulated, whereas the expression levels of α-globulin and glutelins of PB-II were up-regulated [17]. In contrast, we detected no change in the level of α-globulin in the present results (Figure 1b).

Recent studies suggest that chaperone proteins in the ER lumen play a crucial role in the folding and assembly of storage proteins during the formation of PBs [14,24–28]. It has been reported that the rice endosperm storage protein mutant 2 (esp2) contains a high level of glutelin precursor and low levels of prolamin, and glutelin acidic and basic subunits, and that while the level of the chaperone binding protein (BiP) was increased, the level of protein disulfide isomerase (PDI) was reduced [25]. In 13 kD Pro-less transgenic rice the BiP and PDI protein levels were up-regulated relative to the WT, but calnexin (CNX) levels were almost constant [17]. To confirm the changes in expression of chaperone proteins in 13 kD pro-RNAi lines, we analyzed transcript levels in immature seeds at two weeks after flowering using qRT-PCR with the specific primer pairs. BiP, PDI, and CNX were up-regulated relative to the WT in the 13 kD pro-RNAi lines (Figure 1c). These results suggest that abnormal expression levels of seed storage protein genes lead to changed chaperone protein levels in the ER lumen.

### 2.2. Storage Protein Profiles of Transgenic Rice Seeds

**Figure 2:**
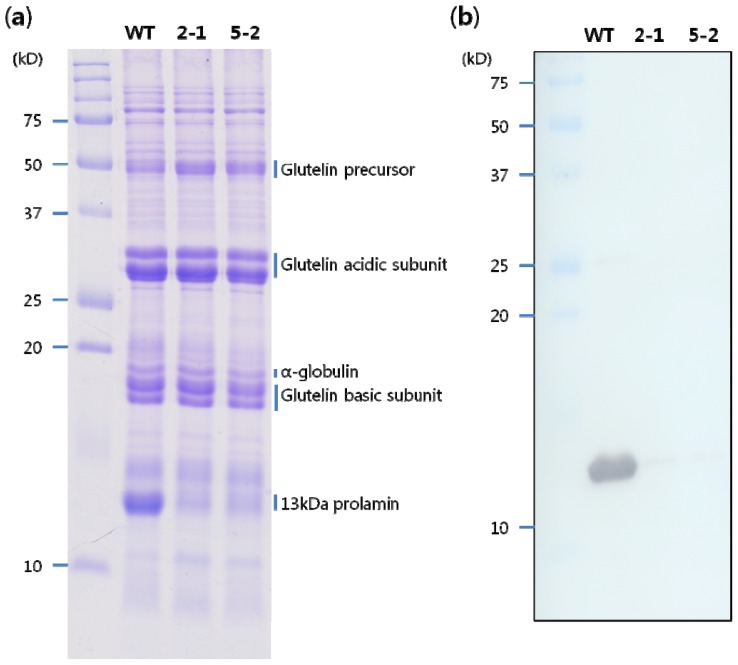
Accumulation of seed storage proteins in mature seeds, (**a**) SDS-PAGE analysis. Total proteins extracted from mature seeds of the wild type (WT) and 13 kD pro-RNAi lines (2-1 and 5-2, respectively) were separated on a 15% SDS-PAGE gel and stained with coomassie brilliant blue (CBB); (**b**) Western blot analysis of 13 kD prolamin. Seed storage proteins resolved by SDS-PAGE were transferred to a polyvinylidenefluoride (PVDF) membrane and incubated with 13 kD prolamin antibody.

The rice seed storage proteins consist of 57 kDa glutelin precursors, 40 kDa acidic and 20 kDa basic glutelin subunits, a 26 kDa α-globulin polypeptide; and 16, 13, and 10 kDa prolamin polypeptides [13]. To analyze changes in storage proteins in the 13 kD pro-RNAi lines the total seed protein was extracted from T_1_ mature seeds and separated by SDS-PAGE. The level of accumulation of 13 kD prolamin in two 13 kD pro-RNAi lines showed significant reduction. However, the level of the glutelin precursor was slightly increased, whereas levels of the remaining storage proteins were not changed in any transgenic line relative to the WT (Figure 2a).

To obtain antibody against the 13 kD prolamins, we purified the 13 kD prolamin proteins using preparative SDS-PAGE. The eluted proteins were used to raise polyclonal antibodies in experimental rats. Western blot analysis was then performed to evaluate the accumulation profile of the 13 kD prolamin. The 13 kD prolamin antibody exhibited strong reactivity to 13 kD polypeptides, with no detectable cross-reactivity to other proteins in the WT. In the 13 kD pro-RNAi transgenic lines, the accumulation of 13 kD prolamins was significantly decreased, to below detectable levels (Figure 2b). Similar results were obtained in independent transgenic lines.

### 2.3. Total Amino Acid Profiles of Transgenic Rice Seeds

**Figure 3:**
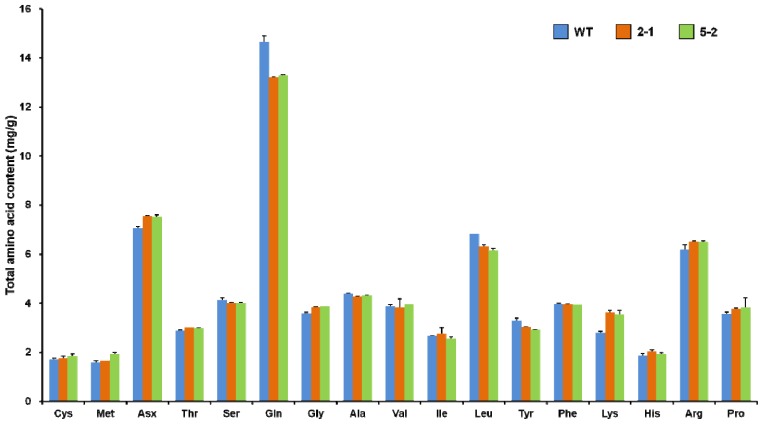
Amino acid composition in mature grains of the wild type (WT) and 13 kD pro-RNAi lines (2-1 and 5-2, respectively). Values are means ± SD (error bars) of two replicates.

Rice is an important protein source for humans and animals. Rice prolamin is indigestible and reduces the protein nutritional quality of rice [29]. Prolamins have a higher content of proline and glutamine, which account for approximately 22% of the total amino acid content [19]. The 13 kD prolamin polypeptides are rich in glutamic acid, aspartic acid and leucine, but low in lysine and sulfur-containing amino acids which are essential for human health. However, 10 kD and 16 kD prolamin polypeptides are rich in sulfur-containing amino acids. On the other hand, glutelins contain greater quantities of lysine than do the prolamins. Because prolamin and glutelin represent a substantial portion of the total storage protein in rice seeds, the ratio of glutelin and prolamin is related to the nutritional quality of seeds [17,30]. As a reduction in prolamin was compensated for by an increase in glutelins (Figure 2a), 13 kD pro RNAi rice protein should be more nutritious. To further investigate the effect of suppressing 13 kD prolamins on the amino acid composition of total seed storage proteins, we determined the total amino acid content of mature seeds of WT and transgenic lines (Figure 3). In the 13 kD pro-RNAi lines the total amino acid content was similar to that in the WT (74.8–75.5 mg/g). However, the level of Glx (glutamic acid and glutamine) was 10% lower, and the level of lysine was 28% higher in the transgenic grains than the wild type. This suggests that 13 kD pro RNAi rice is more nutritious than WT.

### 2.4. PB-I Formation in Transgenic Rice Seeds

Two types of protein bodies (PB-I and PB-II) have been reported to occur in the sub-aleurone layer of rice endosperm [13]. The spherical protein bodies (PB-Is) are rich in prolamin, and the large amorphous protein bodies (PB-IIs) are rich in glutelin [7,13]. During PB-Is formation in the rough ER, the 10 kD prolamin forms the central core and interacts with other cysteine-rich prolamins to assemble a concentric ring structure in the PB-I [18,31]. The 13-I, 13-III, and 16 kD prolamins are cysteine-rich and localized in the middle layer within the PB-I. The cysteine-poor 13 kD (13-II) prolamins are localized in the inner layer surrounding the core and the outermost layer of the PB-I [31]. Glutelins are localized in the inner region of the PB-II, and α-globulin is sequestered in the matrix surrounding the glutelins [12,17].

**Figure 4:**
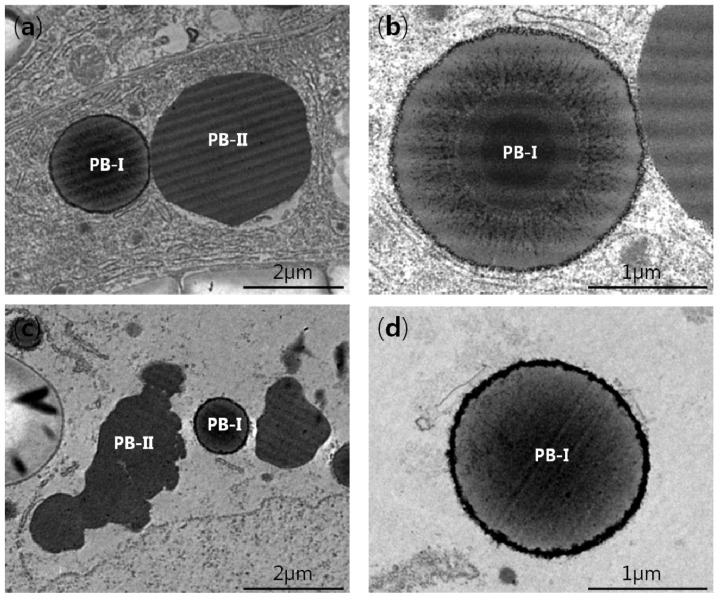
Transmission electron microscopy images of sub-aleurone layer cells at two WAF for the wild type (WT; **a** and **b**) and the 13 kD pro-RNAi line 5-2 (**c** and **d**). PB-I is spherical and had a lamellar structure which is rich in prolamins. In contrast, PB-II is irregular with no lamellar structure which is rich in glutelins and α-globulin.

To study the effect of reduced 13 kD prolamins on PB-I formation, subcellular structures in the sub-aleurone layer of endosperm cells were examined by transmission electron microscopy (TEM). PB-IIs were more common than PB-Is in the sub-aleurone layer cells of WT and transgenic rice. In the WT the PB-I was spherical, and had a lamellar structure with a dark stained central core and one or more layers of lightly stained material that were interspersed with a concentric structure of varying electron density. In contrast, PB-II was irregular with no lamellar structure, and stained homogeneously (Figure 4a,b). In the 13 kD pro-RNAi lines, the PBs were smaller and occurred with lower frequency. Notably, the PB-I of the transgenic lines comprised small spherical structures surrounded by a peripheral heavily stained ring, and had a different inner structure when compared with the PB-I of WT (Figure 4c,d). These findings indicate that 13 kD prolamins are required for formation of the internal structure of PB-I in rice endosperm.

## 3. Experimental Section

### 3.1. Plasmid Constructions

To construct the RNAi cassette for suppressing the 13 kD prolamins a 506 bp rice prolamin (Os07g0206500) cDNA was amplified by PCR using the primers 5′-TTT GCT CTC CTT GCT ATT G-3′ and 5′-CAT GAT GAT GCA TGA TT TAT-3′, containing an attB1 or an attB2 sequence to enable the Gateway cloning system [32]. The amplified product was sub-cloned into the pDONR221 vector (Invitrogen, Carlsbad, CA, USA) using a recombination reaction. The RNAi cassette was recombined with the destination vector pANDA-β containing the maize ubiquitin 1 promoter, the nopaline synthase (Nos) terminator, and the bialaphos resistance gene.

### 3.2. Rice Transformation

The binary vector with the RNAi cassette of 13 kD prolamin was introduced into the rice callus using an *Agrobacterium tumefaciens*-mediated method. Briefly, approximately 200 mature seeds of Japonica-type Korean rice (cv. Ilmi) were husked and sterilized with 70% (*v*/*v*) ethanol for 1 min with gentle shaking. The ethanol was discarded and the seeds were sterilized further with 100 mL of 50% (*v*/*v*) commercial bleach for 40 min with gentle shaking at 180 rpm. The sterilized seeds were then washed several times with sterile water and dried on autoclaved Whatman paper (3 mm) for 5 min. Callus induction, infection with *A. tumefaciens*, and the selection of transformed calli were carried out using the method described by Hiei *et al.* [33].

### 3.3. RNA Extraction and Quantitative Real-Time PCR (qRT-PCR)

Total RNA was extracted from immature seeds at two WAF using Trizol Reagent (Gibco-BRL). Following RNase-free DNase (Invitrogen) treatment the cDNA was synthesized from 2 μg of total RNA using the SuperScript III First-Strand Synthesis System (Invitrogen), according to the manufacturer’s instructions. qRT-PCR was performed in a volume of 20 μL using the AccuPower GreenStar qPCR Master Mix (Bioneer, Korea) and the CFX real-time PCR system and system software (Bio-Rad, USA), according to the manufacturer’s protocol. Each reaction contained 2 μL diluted cDNAs, 10 μL of GreenStar qPCR Master Mix, and 1 μL of each primer. The PCR conditions were as follows: 95 °C for 15 min, followed by 40 cycles of 95 °C for 10 s and 55 °C for 30 s. The melting curves were analyzed at 65–95 °C after 40 cycles. Triplicate reactions were performed and the expression levels were normalized to ubiquitin, using the expression levels of the WT as the reference. The primers used for qRT-PCR are listed in Table S1.

### 3.4. Seed Protein Extraction and Immunoblotting

SDS-PAGE and immunoblotting were performed as described by Kim *et al.* [20]. The total seed protein was extracted from rice seeds (200 mg) by shaking overnight at room temperature in protein extraction buffer (4% SDS, 8M urea, 20% glycerol, 5% 2-mercaptoethanol and 250 mM Tris-HCl; pH 6.8). The extracts were centrifuged at 12,000× *g* for 5 min at room temperature, and the aqueous supernatants were collected and quantified using a protein assay kit (Invitrogen). The proteins (50 μg) were separated using 15% SDS-PAGE, and then transferred to a polyvinylidenefluoride (PVDF) membrane (Millipore). Antibody to the 13 kD prolamin was raised in experimental rats using gel-purified preparations of these putative 13 kD prolamin polypeptides. The membranes were blocked in PBST buffer (phosphate buffered saline, 0.05% Tween 20) containing 5% skim milk, and then reacted with primary 13 kD anti-prolamin in the same buffer. Following washing three times with PBST buffer the membranes were incubated with anti-rat IgG alkaline phosphate conjugate secondary antibody (Pierce, USA). Signals were detected using 5-bromo-4-chloro-3-indolyl phosphate (BCIP) and nitroblue tetrazolium (NBT) (Promega), according to the manufacturer’s instructions. The intensity of the bands was quantified using ImageJ software (http://rsbweb.nih.gov/ij/download.html) [34].

### 3.5. Transmission Electron Microscopy (TEM)

Immature seeds at two WAF were fixed for 2 h at 4 °C in 0.1 M phosphate buffer containing 2.5% glutaraldehyde (pH 7.3). The samples were postfixed on ice for 1 h in 1% osmium tetroxide in 0.1 M phosphate buffer, dehydrated in an ethanol series, and embedded in Epon 812. Ultra-thin sections (80 nm) were cut using an ultramicrotome (Leica Ultracut UCT, Austria) with a diamond knife (Diatome, Switzerland), and stained with uranyl acetate for 18 min followed by lead citrate for 8 min. Thin sections were examined using a LEO 912 AB TEM (Carl Zeiss, Germany) at an accelerating voltage of 120 kV.

### 3.6. Determination of Amino Acid Content

Approximately 300 mg of bulked rice powder was hydrolyzed with 30 mL of 6 N HCl. The mixture was flushed with N_2_ gas for 1 min, dried under vacuum at 110 °C for 24 h, and the residue was dissolved in 0.1 N HCl. For cysteine and methionine analysis, the bulked rice powder was treated with performic acid at 4 °C for 12 h prior to HCl hydrolysis. Amino acids were analyzed using an amino acid autoanalyzer (L-8500A, Hitachi, Hitachinaka, Japan) with a pre-packed ion exchange column, according to the manufacturer’s instructions (Hitachi, Ltd., Tokyo, Japan). Seventeen amino acids were measured (two replicates) in assessing the amino acid content, including: cysteine (Cys), methionine (Met), aspartic acid + asparagine (Asx), threonine (Thr), serine (Ser), glutamic acid + glutamine (Glx), glycine (Gly), alanine (Ala), valine (Val), isoleucine (Ile), leucine (Leu), tyrosine (Tyr), phenylalanine (Phe), lysine (Lys), histidine (His), arginine (Arg), and proline (Pro).

## 4. Conclusions

The 13 kD prolamins, which are indigestible proteins, are the most abundant prolamin group among rice storage proteins. We generated transgenic rice plants (13 kD pro-RNAi) containing RNAi constructs against 13 kD prolamins. Analysis of the transcript levels of the 13 kD pro-RNAi lines showed that the targeted endogenous 13 kD prolamin genes were remarkably suppressed, but the 10 kD prolamin, glutelins and ER chaperone genes were significantly upregulated relative to the WT. The accumulation of the 13 kD prolamins in transgenic seeds was not detected by immunoblot analysis using 13 kD prolamin antibody. In particular, the reduction in 13 kD prolamins at the mRNA and protein levels resulted in 28% increase of the level of lysine, and abnormal formation of PB-I in the transgenic grains. These results indicate that the 13 kD pro-RNAi rice seeds are more nutritious than the WT, suggesting that regulation of endogenous storage protein content by RNAi is a promising tool for improvement of the nutritional quality of rice grains.

## Supplementary Materials
