# Supplementary material for: Effects of Reduced Prolamin on Seed Storage Protein Composition and the Nutritional Quality of Rice
Source: Int J Mol Sci. 2013 Aug 19;14(8):17073–84. doi: 10.3390/ijms140817073 (PMC3759952; doi:10.3390/ijms140817073)
Supplement: Supplementary file 1 [file ijms-14-17073-s001.pdf]

Supplementary Materials

Table S1. Genes and primer sequences used for qRT-PCR.

Target Gene	Primer name	Forward primer	Reverse primer	Accession No.
**Prolamin 10 kD**	10 kD	TTATTTGTGCTGGACTCGGG	GAGAGTTGGAAGTTGACAGGG	EF122448
**Prolamin 13 kD Class I**	13 kD-I	CAACTACAGTCGCATCTCCTAC	GGGTTGCCACTATGCTATACTG	EF122447
**Prolamin 13 kD Class II**	13 kD-II	GCTCTGTTGGCTTTTAACGTG	ACTCATTACAAGACACCGCC	GU120358
**Prolamin 13 kD Class III**	13 kD-III	TCACCCGTGTTTCAACTGAG	CACAATAGCCTGAACACTGC	FJ940200
**Prolamin 16 kD**	16 kD	CTCAATTTGCCCTCCATGTG	AGAACCGCAATGACCAGTAG	EF122449
**Glutelin A**	GluA	AATGATGGTGAAGTGCCGGT	TCACGCCTGTATGCTTGAGG	EF122456
**Glutelin B**	GluB	ATTGAGCAACACTCTGGGCA	TGGCTCTGTAGCCTCTTTGC	EF122460
**Glutelin C**	GluC	CACAAGGGCCAATAGCCAGA	GGTCACGTACATCACCGTGT	EF122465
**Glutelin D**	GluD	AAGACAGAGCGACCAAGCTC	ATGTGCAACACTAGCCGGAA	EF122464
**Globulin-1**	Glb-1	AGTCGGAGATGAGGTTCAGG	GAACATCGGCTGGAACCTC	GQ848069
**ER luminal binding protein**	BiP	AGGACATCAGCAAGGACAAC	GGACTCAATCTCAACACGGAC	AK065743
**Protein disulfide isomerase**	PDI	CCGATTGTTCTTGCCAAGGTTG	TCCTGAATGTTCTTGCCCTG	AK068268
**calnexin**	CNX	TCGACAACCCCAACTACAAAG	ATCTCAATCCCAATAGCGGC	AK069118
**Ubiquitin**	Ubi	GAAGTAAGGAAGGAGGAGGA	AAGGTGTTCAGTTCCAAGG	EF575840

© 2013 by the authors; licensee MDPI, Basel, Switzerland. This article is an open access article distributed under the terms and conditions of the Creative Commons Attribution license (http://creativecommons.org/licenses/by/3.0/).
